# Supplementary material for: Multipronged Approach to Profiling Metabolites in Beta vulgaris L. Dried Pulp Extracts Using Chromatography, NMR and Other Spectroscopy Methods
Source: Foods. 2023 Sep 21;12(18):3510. doi: 10.3390/foods12183510 (PMC10528680; doi:10.3390/foods12183510)
Supplement: Supplementary file 1 [file foods-12-03510-s001.zip › foods-2561938-supplementary.pdf]

Supporting Information

Multipronged Approach to Profiling Metabolites in *Beta Vulgaris* Dried Pulp Extract Using Chromatography, NMR, and other Spectroscopy Methods

Joshua Fiadorwu[1], Kiran Subedi[2], Daniel Todd[3], and Mufeed M. Basti[1*]

Affiliation

[1] Department of Applied Science and Technology, College of Science and Technology, North Carolina Agricultural and Technical State University, Greensboro, NC, USA.

[1*]Department of Chemistry, North Carolina Agricultural and Technical State University, Greensboro, NC, USA.

[2]Analytical Services Laboratory, College of Agriculture and Environmental Sciences (CAES), North Carolina Agricultural and Technical State University, Greensboro, NC, USA.

[3] Triad Mass Spectrometry Facility, University of North Carolina at Greensboro, NC, USA.

Correspondence

Mufeed M. Basti

Professor

NC A&T State University

Department of Chemistry

Science Building

Greensboro, NC 27455

Ph. (336)285-2229

basti@ncat.edu

Contents

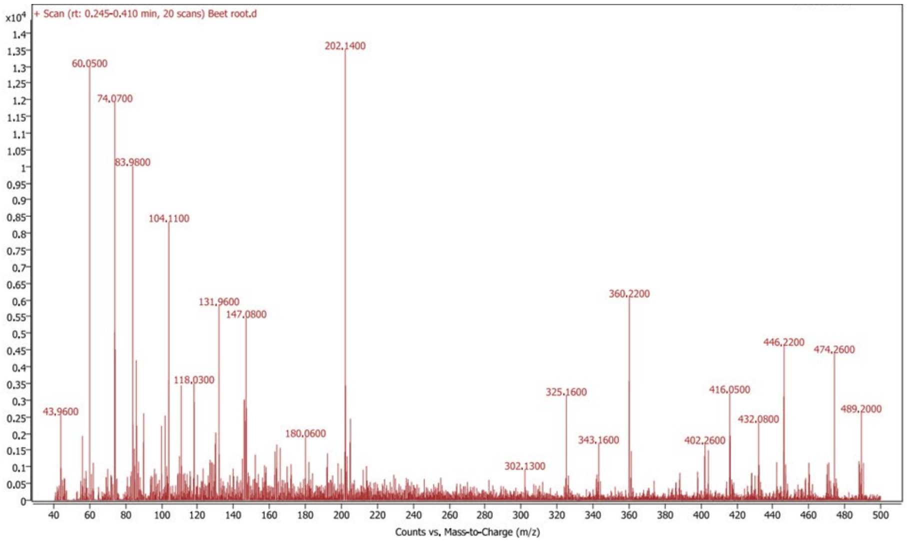

Figure S1. The MS fragmentation spectrum of the compounds and metabolites in the aqueous extract of beetroot powder in positive polarity mode.

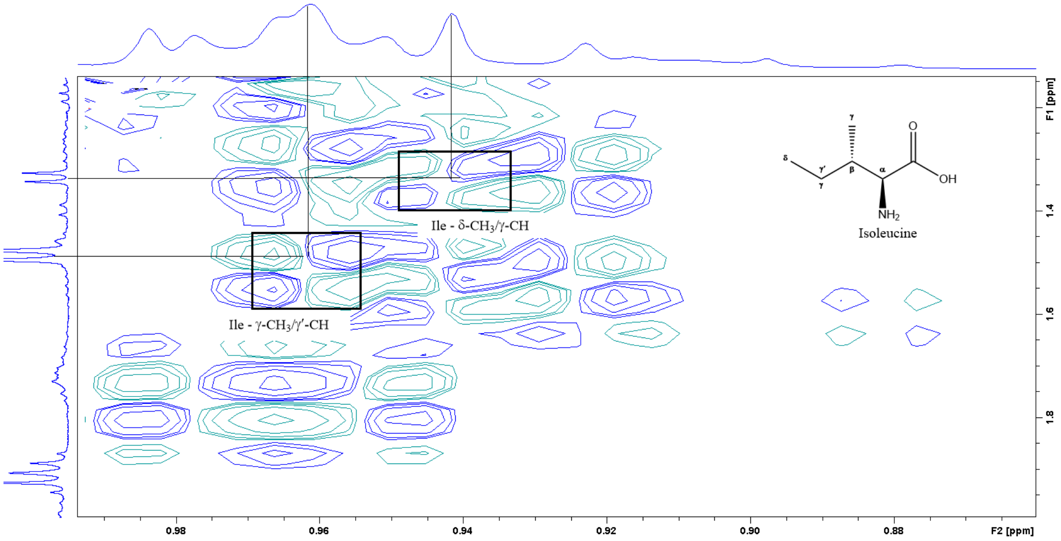

Figure S2. A section of the COSY spectrum showing spin coupling in the amino acid Isoleucine.

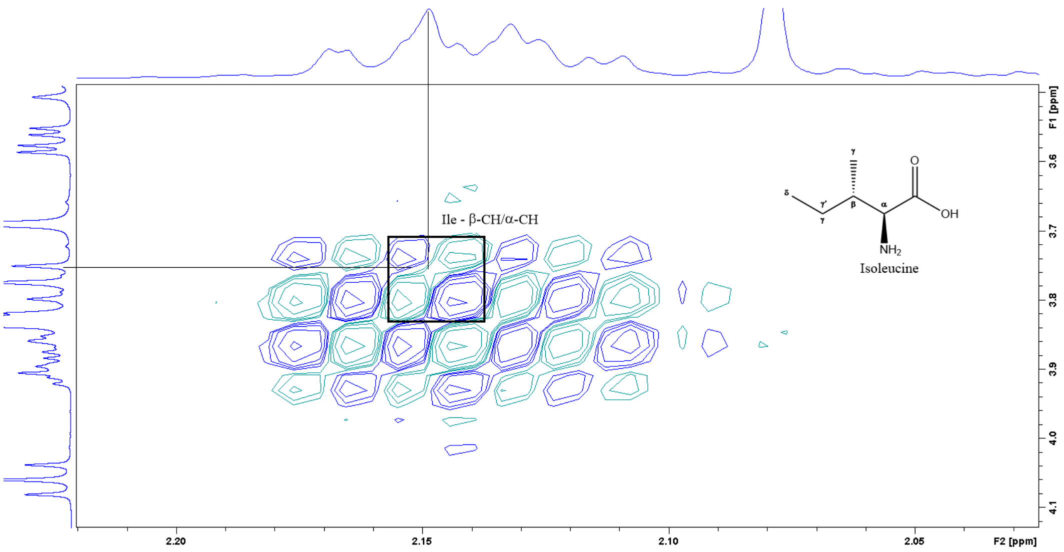

Figure S3. A section of the COSY spectrum showing the amino acid Isoleucine.

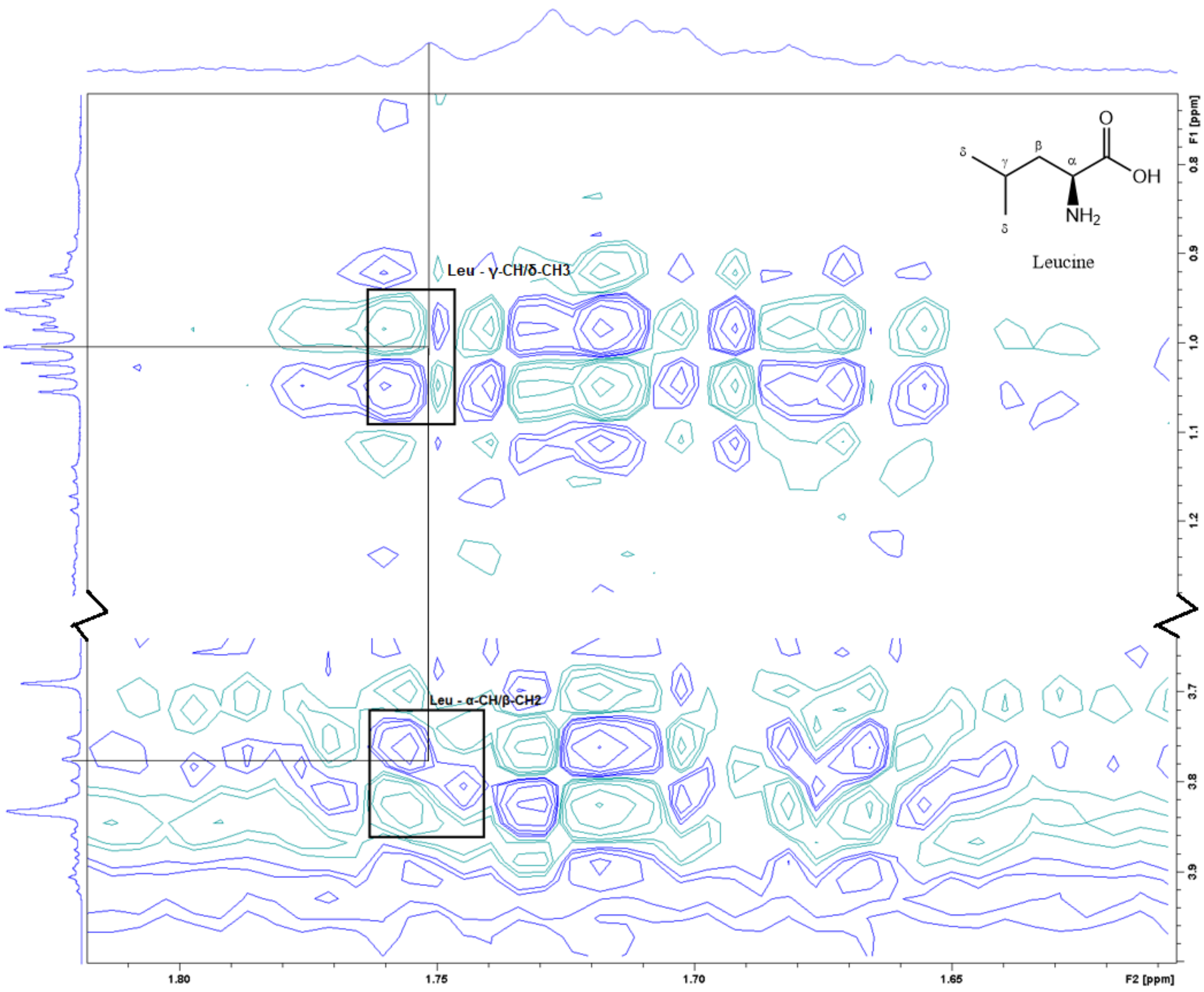

Figure S4. COSY spectrum showing the amino acid Leucine (D_2O-salt, 400 MHz).

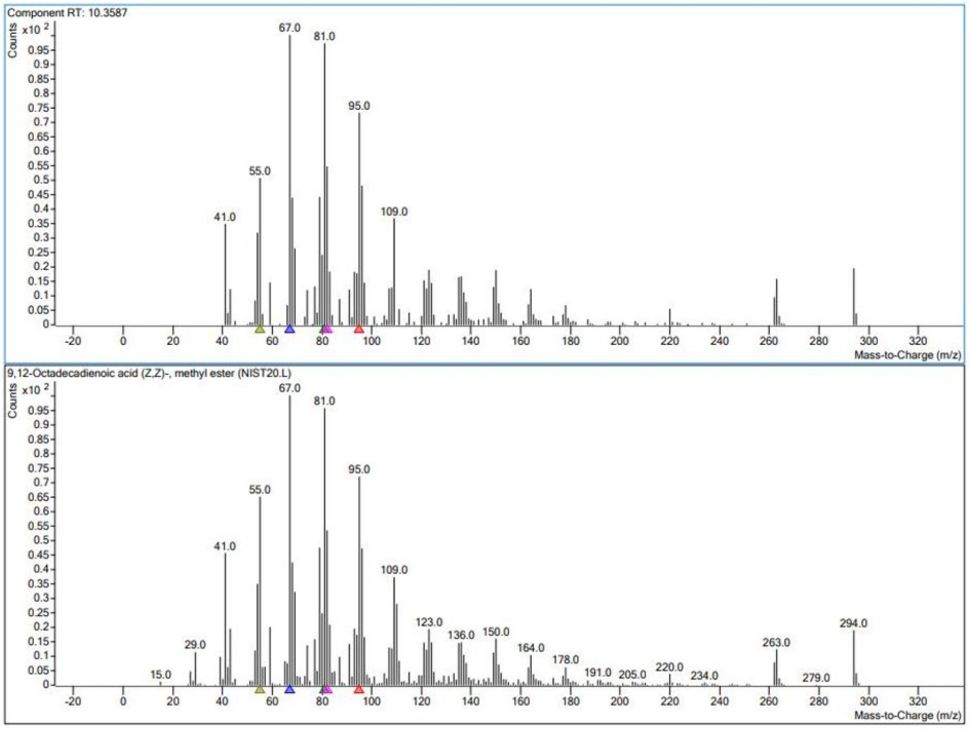

Figure S5. MS spectra of 9,12-Octadecadienoic acid, methyl ester (RT = 15.419 min.) experiment spectrum overlaid with the reference library spectrum.

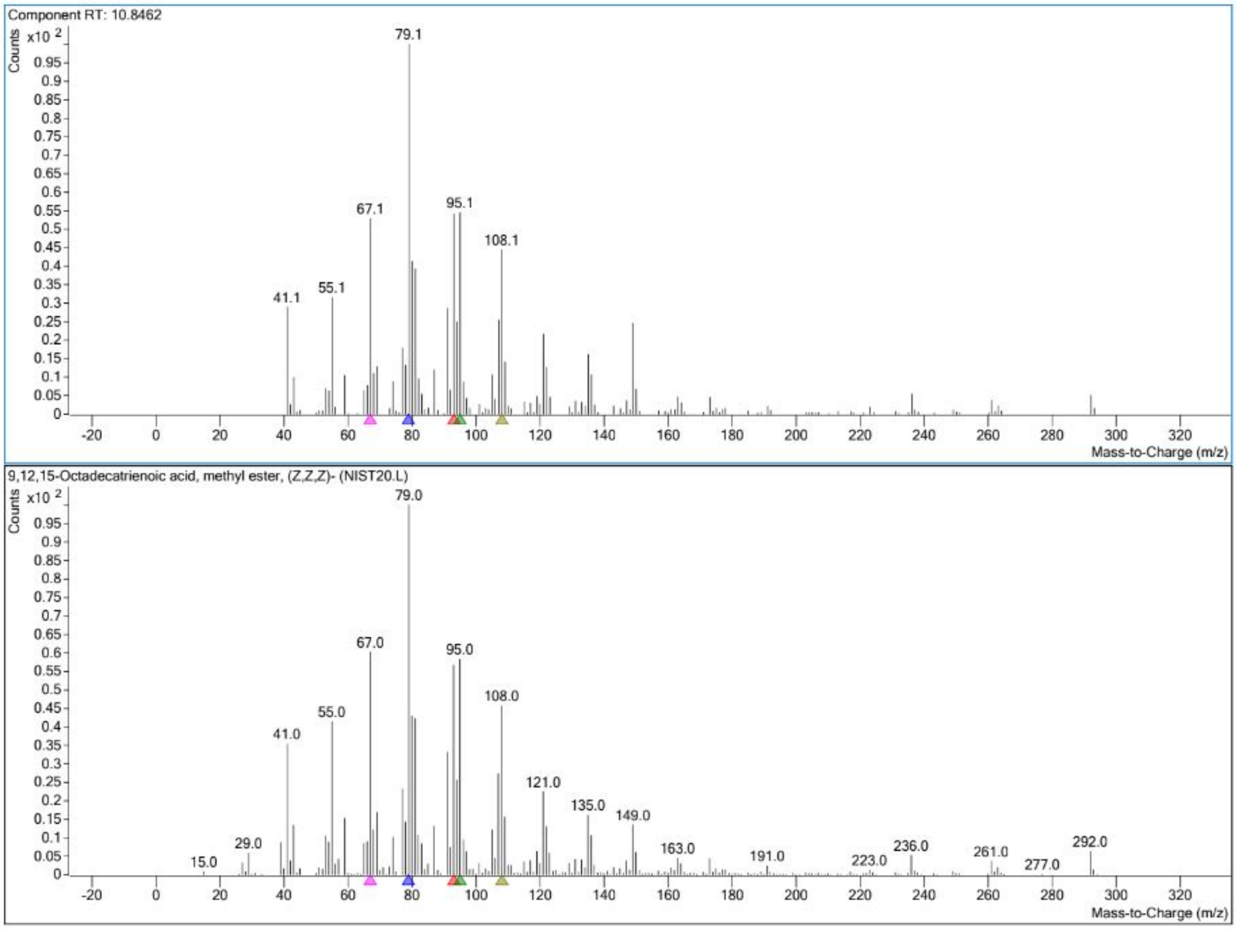

Figure S6. MS spectra of 9,12,15-Octadecatrienoic acid, methyl ester, (Z,Z,Z) (RT = 10.846 min.) experiment spectrum overlaid with the reference NIST library spectrum.

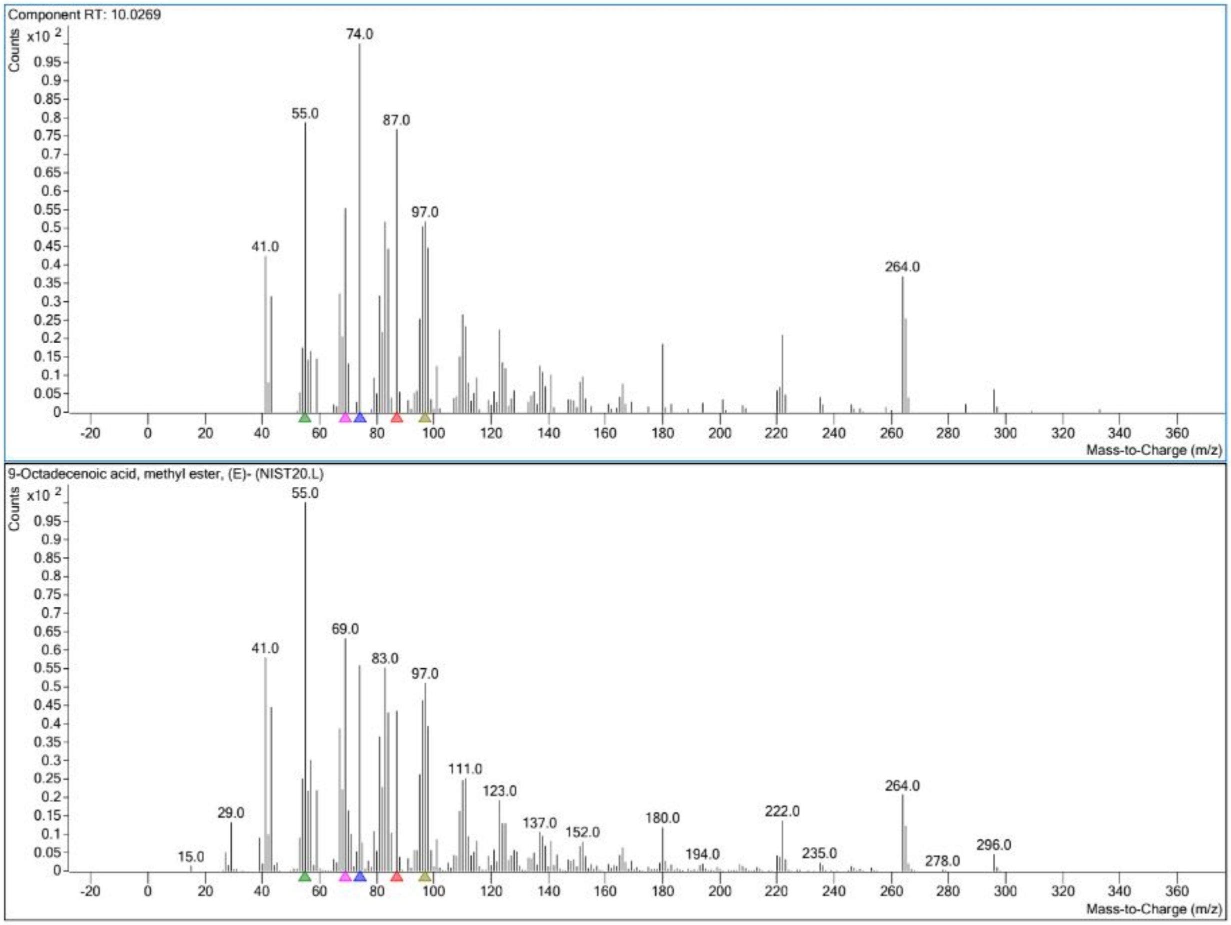

Figure S7. MS spectra of 9-Octadecenoic acid, methyl ester, (E) (RT = 10.027 min.) experiment spectrum overlaid with the reference NIST library spectrum.

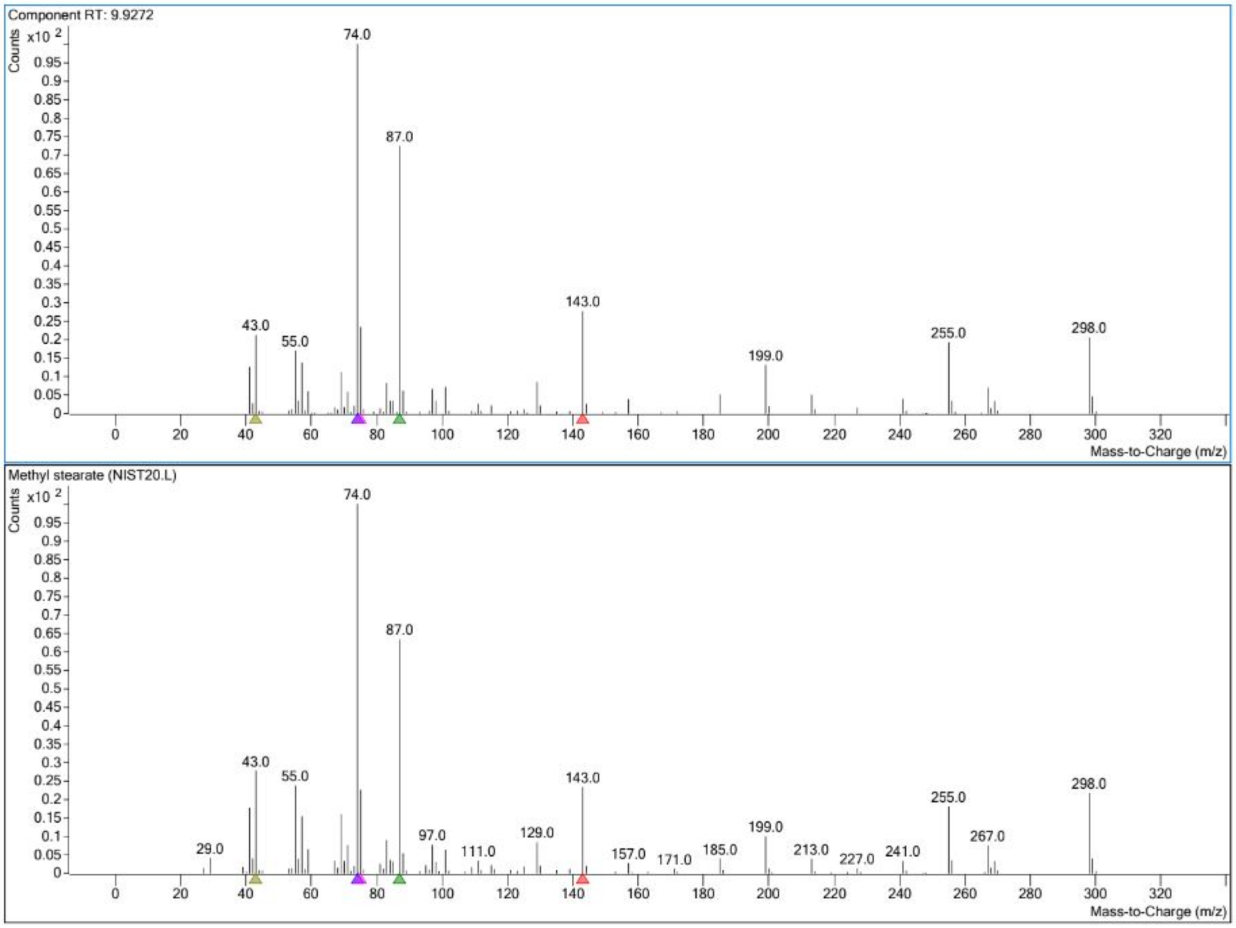

Figure S8. MS spectra of methyl stearate (RT = 9.927 min.) experiment spectrum overlaid with the NIST reference library spectrum.

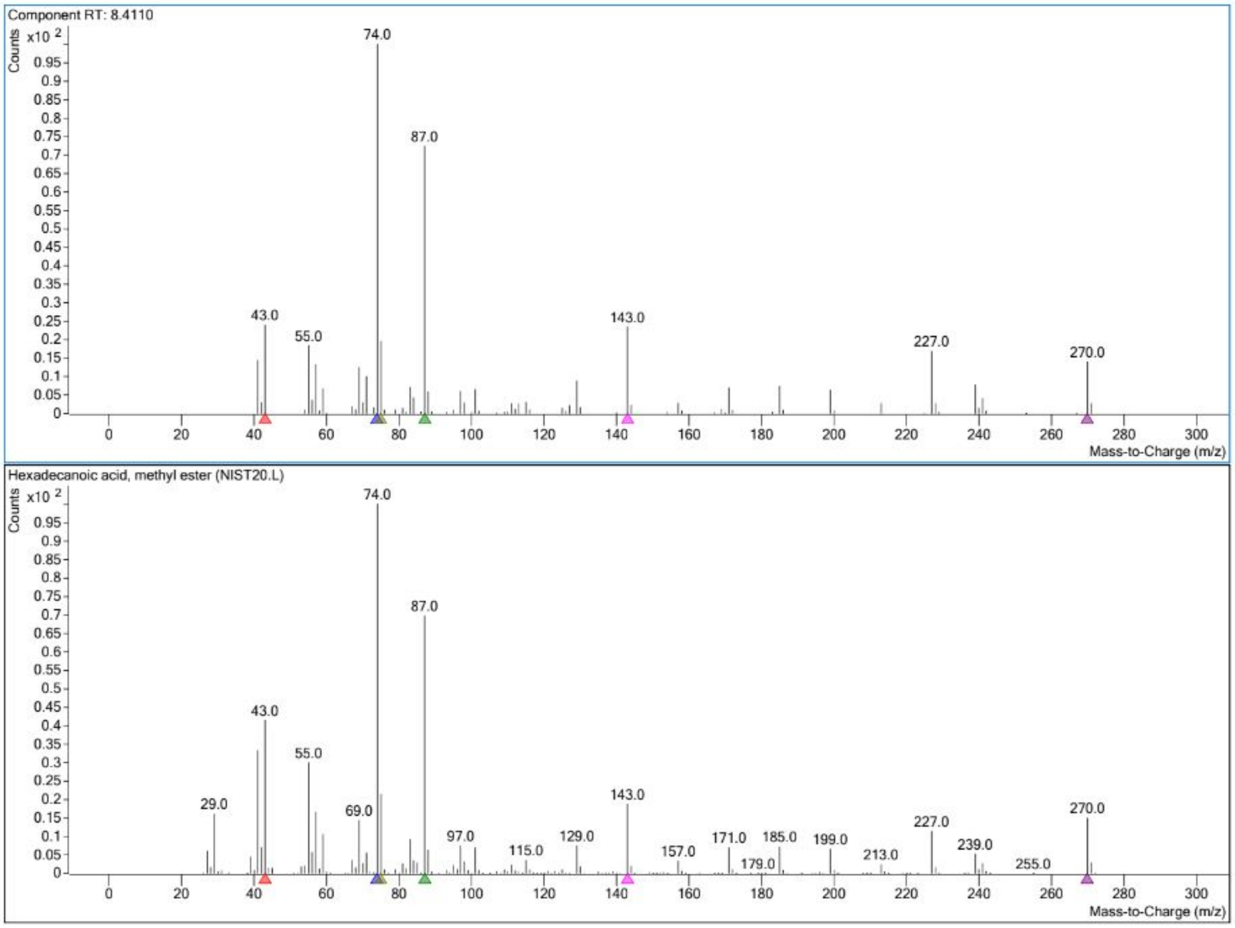

Figure S9. MS spectra of hexadecanoic acid, methyl ester (RT = 8.411 min.) experiment spectrum overlaid with the NIST reference library spectrum.

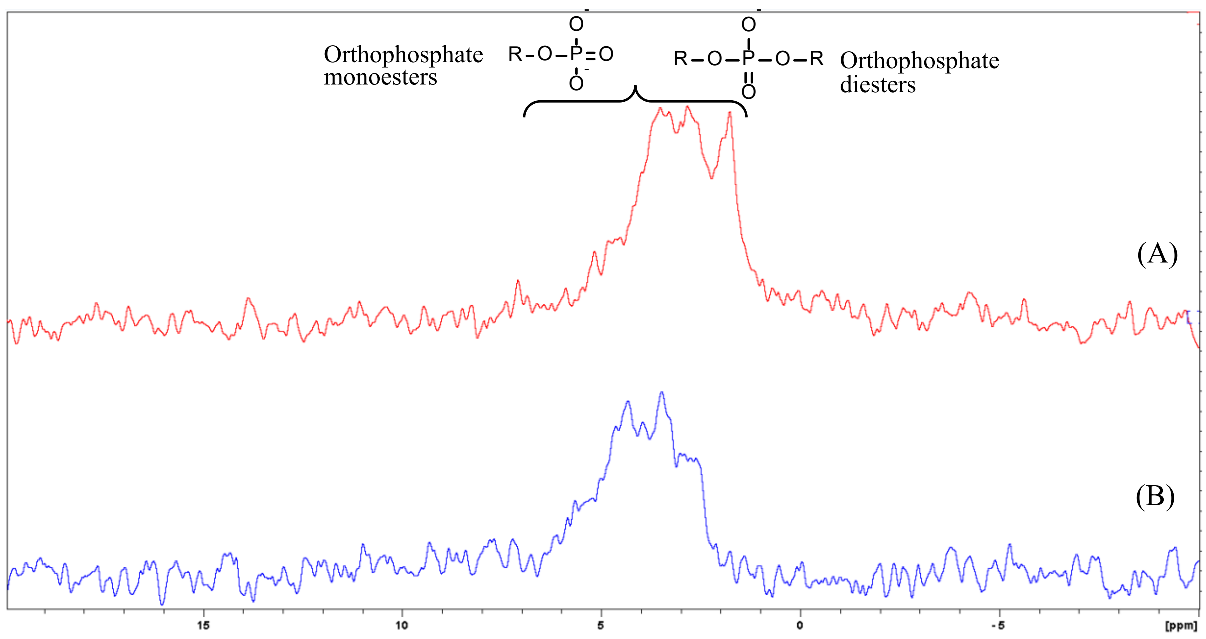

Figure S10. Proton-decoupled ^{31}P NMR spectra of polar extract (A) in D_2O and non-polar extract (B) in $CDCl_3$ on a 400 MHz spectrometer, ns = 10,000 at 25°C temperature, and 20 Hz line-broadening. Chemical shift is based on the 3^{1}P peak of 85% phosphoric acid, which is set at 0 ppm.

$^{13}C/^{1}H$ Atom label (sucrose)	Measured chemical shift, δ (ppm)	Literature chemical shift, δ (ppm) [33]	^{1}H Atom label (α-D-glucopyranose/β-D-glucopyranose)	Measured chemical shift, δ (ppm) of (α-D-glucopyranose/β-D-glucopyranose)	Literature chemical shift, δ (ppm) [34]
C_1/H_1	92.15/5.41	92.15/5.40	H_1	5.25/4.68	5.35/4.74
C_2/H_2	71.13/3.56	71.08/3.55	H_2	3.53/3.39	3.64/3.37
C_3/H_3	72.65/3.77	72.65/3.75	H_3	3.70/3.48	3.81/3.60
C_4/H_4	69.26/3.48	69.26/3.46	H_4	3.56/3.91	3.52/3.92
C_5/H_5	72.09/3.85	72.10/3.82	H_5	3.85/3.51	3.98/3.50
C_6/H_6	60.15/3.81	60.15/3.82			
C'_1/H'_1	61.36/3.68	61.40/3.67			
$C'_3H'_3$	76.45/4.22	76.50/4.21			
C'_4/H'_4	74.05/4.06	74.10/4.04			
C'_5/H'_5	81.35/3.90	81.39/3.89			
C'_6/H'_6	62.43/3.83	62.50/3.82			

Table S1. The chemical shift values, δ (ppm) of the ^{1}H of sucrose and α- and β-D-glucopyranose and the corresponding literature values, and δ (ppm) of ^{13}C of sucrose and the corresponding literature values.

Amino acid	^{1}H Chemical shifts, δ (ppm)	^{1}H Chemical shifts, δ (ppm) Literature values[36]
Leucine	δ-CH_3-0.978<br>γ-CH, β-CH_2-1.765<br>α-CH-3.779	δ-CH_3-0.948<br>γ-CH, β-CH_2-1.700<br>α-CH-3.722
Isoleucine	γ-CH_3-0.942<br>δ-CH_3-0.962<br>γ-CH-1.343<br>γ^{1}-CH-1.493<br>β-CH-2.149<br>α-CH-3.749	γ-CH_3-0.926<br>δ-CH_3-0.997<br>γ-CH-1.248<br>γ^{1}-CH-1.457<br>β-CH-1.968<br>α-CH-3.660
Valine	α-CH-3.689<br>β-CH-2.284<br>γ^{1}-CH_3-1.036<br>γ-CH_3-0.984	α-CH-3.601<br>β-CH-2.262<br>γ^{1}-CH_3-1.029<br>γ-CH_3-0.976

Table S2. ^{1}H chemical shift of identified amino acids and literature values.

Chemical shift (δ) (ppm)	Assigned H
5.36	9,10,12,13,15,16 – Linolenic acid<br>9,10,12,13 – Linoleic acid<br>9,10 – Oleic acid
2.77	11 – Linoleic acid<br>11,14 – Linolenic acid
2.31	2 – α-CH_2
2.06	8,17 – Linolenic acid<br>8,14 – Linoleic acid<br>8, 11 – Oleic acid
1.60	3 – β-CH_2
1.26	* - CH_2(s) (overlapping)
0.88	18 - CH_3 (terminal)

Table S3. Chemical shift values and assigned protons in the selective TOCSY spectrum. The asterisk (*) indicates the overlapping CH_2 groups.
